# The role of body temperature on respiratory rate in children with acute respiratory infections

**DOI:** 10.4314/ahs.v21i2.20

**Published:** 2021-06

**Authors:** Beril Ozdemır, Sıddıka Songül Yalçın

**Affiliations:** 1 Department of Pediatrics, Baskent University Faculty of Medicine, Ankara, Turkey; 2 Department of Pediatrics, Hacettepe University Faculty of Medicine, Ankara, Turkey

**Keywords:** Fever, tachypnea, pneumonia, respiratory rate difference, children

## Abstract

**Background:**

The World Health Organization (WHO) recommends the use of tachypnea as a proxy to the diagnosis of pneumonia.

**Objective:**

The purpose of this study was to examine the relationship between body temperature alterations and respiratory rate (RR) difference (RRD) in children with acute respiratory infections(ARI).

**Methods:**

This cross-sectional study included 297 children with age 2–60 months who presented with cough and fever at the pediatric emergency and outpatient clinics in the Department of Pediatrics, Baskent University Hospital, from January 2016 through June 2018. Each parent completed a structured questionnaire to collect background data. Weight and height were taken. Body temperature, respiratory rate, presence of the chest indrawing, rales, wheezing and laryngeal stridor were also recorded. RRD was defined as the differences in RR at admission and after 3 days of treatment.

**Results:**

Both respiratory rate and RRD were moderately correlated with body temperature (r=0.71, p<0.001 and r=0.65, p<0.001; respectively). For every 1°C increase in temperature, RRD increased by 5.7/minutes in overall, 7.2/minute in the patients under 12 months of age, 6.4/minute in the female. The relationship between body temperature and RRD wasn't statistically significant in patients with rhonchi, chest indrawing, and low oxygen saturation.

**Conclusion:**

Respiratory rate should be evaluated according to the degree of body temperature in children with ARI. However, the interaction between body temperature and respiratory rate could not be observed in cases with rhonchi and severe pneumonia.

## Introduction

Acute Respiratory Infections (ARI) is a major cause of child mortality among children under 5 years, worldwide^1^,^2^. The World Health Organization (WHO) global report considered that pneumonia accounts for approximately 120 million cases every year, among which 14 million (12%) progress to severe pneumonia in 2013^3^. ARIs in young children present with nonspecific complaints such as fever, cough, and poor feeding. Clinical examination can reveal tachypnea, nasal flaring, grunting, chest indrawing, cyanosis, abnormal breath sounds (rales, rhonchi)^2^, ^4^.

Fever is a common symptom in children and occurs as an adaptive response to inflammation that results from infection^5^, ^6^. Fever is defined as a regulated increase in body temperature above the normal thermal set point in response to inflammation1. High fever, typically defined as 39.5°C or greater, has been associated with increased mortality in critically ill patients^7^. Fever is a remarkable sign of the acute phase response to infectious and noninfectious sources of tissue injury, so fever is common in patients with ARI. Alterations in body temperature with hypoalbuminemia and ambient temperatures were reported8.The relationship between body temperature alterations and respiratory differences in ARI and outcomes is not well known. Understanding this relationship may provide evidence for fever suppression or warming interventions. Also defining the role of body temperature changes on respiratory rate may contribute to diagnostic evaluations and treatment of cases.

The purpose of this study was to examine respiratory rate (RR) changes by body temperature alterations during respiratory tract infection in children.

## Materials and Methods

### Study subjects

This cross-sectional study included 297 children with age 2–60 months having complaints of cough with or without fever at the pediatric emergency and outpatient clinics in the Department of Pediatrics, Baskent University Hospital, from January 2016 through June 2018. Children having any antipyretics within the last four hours were not included in the study.

The study conformed to the principles outlined in the Declaration of Helsinki. Informed written consent was obtained from the parents of children. The study was approved by Başkent University Institutional Review Board (Project no:KA18/299).

### Study design

One parent (mother or father) completed a structured questionnaire to collect data regarding the child's age and gender. In addition, a history of low birth weight, prematurity, delivery type, presence of a history of chronic disease, and pneumonia were collected, weight, and height of children were taken. Physical examination was performed and body temperature, RR, presence or absence of the chest indrawing, rales, wheezing, and laryngeal stridor were recorded on admission. Patients were re-evaluated, and respiratory rate and body temperature were taken at the following two days (on 1^st^ day, 2^nd^ day of treatment) and 1 week after recruitment.

In each visit, RR counted for a minute by observing the chest movements, with the child lying down and without crying^9^,^10^. RR difference (RRD) was defined as the respiratory rate difference between data after recovery and data with the first three days of illness. Chest retractions were recorded if intercostal or subcostal retractions were present^11^,^12^.

Body temperature was measured from the tympanic site^13^ and recorded for up to 7 days. All temperature measurements were conducted by the same experienced pediatric nurse. We measured tympanic temperature in the right ear as a standard measurement by a thermometer (Braun ThermoScan® ExacTempTM IRT4520, Lausanne, Switzerland). Each tympanic measurement was repeated 2 times and the mean of the 2 values was accepted as body temperature. The calibration of the thermometer was checked regularly, two times in a day.

### Classifications

We defined “pneumonia” in a child with a cough or has difficulty in breathing or any of the danger signs such as inability to feed, lethargy, central cyanosis, or grunt; RR≥ 50/min in infants up to 12 months of age and RR≥40/min in children older than 12 months; and a suggestive radiograph^14^. “Bronchiolitis” was defined as the first episode of wheezing with evidence of an acute viral respiratory tract infection (coryza), an axillary temperature of ≥37.8°C, cough, predominant wheeze, or rhonchi on chest auscultation and suggestive radiograph^14^. And a child with cough or difficult breathing but none of the signs as chest indrawing, stridor is classified as “cough or cold”.

We divided weight by the squared height to calculate BMI (in kg/m^2^). Using WHO Anthro, z scores for body mass index-for-age, weight for age, height for age, and weight for height were calculated^11^.

### Statistical analysis

Data analysis was performed with SPSS (Statistical Package for Social Sciences, Version 22, SPSS Inc., Chicago, IL). The data were expressed as the number of observations, percentages, mean ± standard deviation, and median. The normality of data was evaluated by Kolmogorov Smirnov test, histogram, skewness, and kurtosis tests. The correlation between body temperature and RR parameters (RR andRD on admission, Day 1, and Day 2) were evaluated with Pearson or spearman's correlation test where appropriate. Linear regression analysis tested the relationship between RR parameters (RR and RRD on admission) and fever, B Coefficients with 95 % CI were given. P<0.05 was considered significant.

## Results

The clinical characteristics of the study subjects are shown in Table 1. The mean (median) age of 297 patients was 25.8 (24.0) months. 49.2% of the study subjects were male (Table 1). There were 114 patients with cough complaints and 183 patients with fever and cough (Table 1). Overall, 19.9% (n = 59) of the cases were diagnosed as cough or cold, 39.4% (n = 117) were diagnosed as pneumonia and 40.7% (n = 121) were diagnosed as bronchiolitis (Table 1).

**Table 1:** Baseline characteristics of the patients, n=297

Parameters		Mean±SD (median)	N (%)
Age	month	25.8±16.7	
Age	<12 month		79 (26.6)
	≥12 month		218 (73.4)
Sex	Female		151 (50.8)
	Male		146 (49.2)
Delivery type	Vaginal		155 (52.2)
	Cesarean		142 (47.8)
Low birth weight	Absence		240 (80.8)
	Presence		57 (19.2)
Prematurity	Absence		229 (77.1)
	Presence		68 (22.9)
Chronic disease history	Absence		221 (74.4)
	Presence		76 (25.6)
Past history of lower respiratory tract infection	Absence		195 (65.7)
	Presence		102 (34.3)
Complaint on admission	Cough alone		114 (38.4)
	Cough and fever		183 (61.6)
Diagnosis	Cough or cold		59 (19.9)
	Pneumonia		117 (39.4)
	Bronchiolitis		121 (40.7)
Weight for age	Z score	0.33±0.95	
		(0.42)	
Height for age	Z score	0.55±1.28	
		(0.53)	
Weight for height	Z score	0.05±1.11	
		(0.14)	
Body mass index for age	Z score	-	
		0.02±1.13	
		(0.06)	
Body mass index for age	<-1 z score		57 (19.2)
	≥-1 and ≤1		190 (64.0)
	z score		50 (16.8)
	>1 z score		

**Figure 1:**
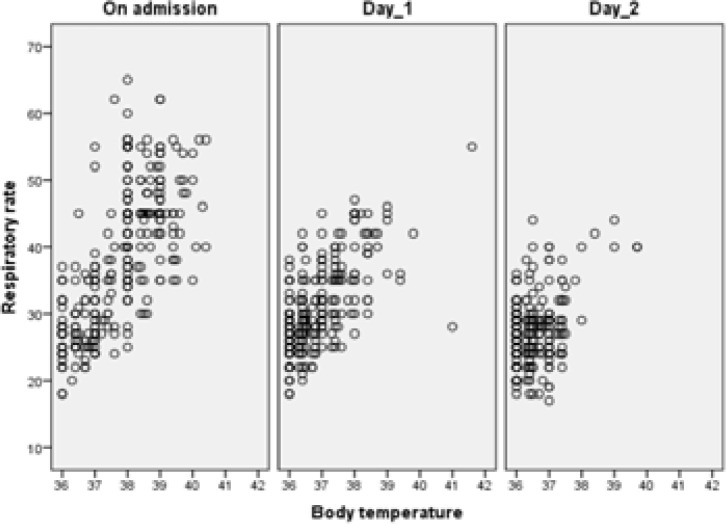
Changes in respiratory rate with body temperature during follow-up period

It is observed that when body temperature is increased, respiratory rate and RRD are also increased (Fig 1). A moderate correlation was detected between them on admission. However, correlations between body temperature and RRD decreases with treatment (r=0.65 p<0.001 on admission, r=0.54 p<0.001 for the first day and r=0.08 p>0.05 for the second day, Table 2).

**Table 2:** Changes in body temperature and respiratory rate during follow-up period and correlation co-efficients between them, n=297

	On admission	1^st^ day of treatment	2^nd^ day of treatment	After treatment
Body temperature, °C*	37.7±1.1 (38.0)	36.8±0.9 (36.5)	36.5±0.6 (36.4)	
Respiratory rate, per min*	38.0±10.4 (37.0)	30.6±6.2 (29.0)	27.2±4.7 (27.0)	25.5±3.6 (25.0)
RRD*	12.8±10.0 (11.0)	5.3±6.6 (4.0)	2.0±5.0 (1.0)	
Correlations&				
Body temperature and respiratory rate	0.71#	0.63#	0.18#	
Body temperature and RRD	0.65#	0.54#	0.08	

RRD: respiratory rate difference between the data after recovery and the data with the first three days of illness

* Mean±SD (Median)

& Pearson correlation coefficients were used for data on admission, Spearman's correlation coefficients for data on Day 1 and Day 2.

# p<0.01

The effect of clinical characteristics of the patients on respiratory difference is shown in Table 3. For every 1°C increase in temperature, respiratory rate rise by 6.5/minute and RRD by 5.7/minute (p<0.001 for both parameters; Table 3). These differences were detected regardless of age, sex, body mass index for age z score, type of delivery, birth weight and presence of chronic disease. Change in RRD with body temperature was observed to decrease faster in patients under 12 months (for each 1°C change 7.2/min for cases <12 months, 5.6/min ≥12 months) (Table 3).

**Table 3:** Changes in respiratory rate and RRD (respiratory rate difference after recovery with admission value) with body temperature according to baseline characteristics of patients, n=297

		Respiratory rate with body temperature	RRD with body temperature
Overall		6.55.8; 7.2**	5.75.0; 6.5**
Age, months	<12	8.26.4; 9.9**	7.25.4; 91**
	≥12	6.55.6; 7.3**	5.64.7; 6.5**
Sex	Female	7.16.0; 8.1**	6.45.3;7.5**
	Male	5.84.8; 6.9**	4.93.8; 6.0**
Body mass index, z score	<-1	6.45.1; 7.7**	5.94.5; 7.4**
	≥-1 and ≤1	6.65.6; 7.6**	5.84.7; 6.8**
	>1	6.44.4; 8.4**	5.13.1; 7.1**
Complaint on admission	Cough, alone	5.43.9; 6.9**	5.23.7; 6.8**
	Fever and cough	5.03.6; 6.4**	3.82.4; 5.2**
Chest auscultation	No	3.92.4; 5.4**	4.73.0; 6.4**
	Rales	3.72.4; 5.0**	3.01.5; 4.4**
	Rhonchi	3.0–1.0; 7.0	3.1–1.2; 7.5
	Rales and rhonchi	3.51.3; 5.7*	3.61.3; 5.9*
Retraction on admission	Absence	4.74.0; 5.4**	4.03.2; 4.7**
	Presence	0.7–1.1; 2.5	0.2–1.9; 2.2
Saturation on admission	<95%	2.0–0.2; 4.2	1.5–1.0; 3.9
	≥95%	5.24.8; 5.8**	4.53.7; 5.2**
Diagnosis on admission	Cough or cold	0.8–2.2; 3.8	1.7–1.7; 5.0
	Pneumonia	4.63.8; 5.4**	3.92.9; 4.8**
	Bronchiolitis	3.71.9; 5.5**	3.51.5; 5.5*
Past history			
Delivery type	Vaginal	6.25.2; 7.2**	5.54.5; 6.5**
	Cesarean	6.95.8; 8.1**	6.04.8; 7.2**
Low birth weight	Absence	6.45.6; 7.2**	5.64.8; 6.5**
	Presence	5.83.7; 7.8**	4.92.6; 7.2**
Prematurity	Absence	6.55.6; 7.5**	5.84.9; 6.7**
	Presence	6.65.3; 7.9**	5.64.1; 7.1**
Chronic disease history	Absence	6.25.4; 7.1**	5.64.7; 6.5**
	Presence	7.15.7; 8.6**	6.14.4; 7.7**
Past history of pneumonia	Absence	6.65.7; 7.5**	5.84.8; 6.8**
	Presence	5.33.5; 7.1**	4.62.7; 6.4**

Linear regression analysis, Unstandardized coefficients (B)95 % CI

* p<0.05

** p<0.001

There is no relation between respiratory rate parameters, and body temperature in patients having retraction at admission (B95 % CI: 0.7–1.1;2.5 for respiratory rate and 0.2–1.9; 2.2 for RRD) (Table 3).

Chest auscultation affects the relationship between body temperature and RRD in children with ARI. For every 1°C increase in temperature, RRD increased by 4.7/minute95% CL 3.0; 6.4, p<0.001 in cases having neither rales nor rhonchi and 3.095% Cl 1.5; 4.4, p<0.001 in cases having rales. However, respiratory rates and RRD in patients having rhonchi and having both rales and rhonchi were not related to body temperature on admission.

Oxygen saturation of patients influenced the relationship between respiratory rate parameters and body temperature; the interaction between them was disappeared in patients having oxygen saturation lower than 95% (Table 3).

## Discussion

This is the first study to evaluate the relationship between body temperature and RR parameters in children with ARI. RR was detected to be influencedby body temperature.

In our study, the presence of rales with or without rhonchi affected the RRD with body temperature. Previous studies have evaluated physical examination findings in the diagnosis of pneumonia in children^15^,^16^. March and Sant'Anna observed that the presence of tachypnea had a sensitivity of 77% and a specificity of 39% for the detection of pneumonia among children less than 6 months of age^17^. Falade et al. also found tachypnea to be 79% sensitive and 65% specific in the diagnosis of pneumonia among children less than 5 years of age^18^. Previously, regardless of age, BMI, sex, birth weight, chronic disease history, and history of lower respiratory tract infection tachypnea were reported to be usefuln distinguishing children with pneumonia^16^. However, body temperature did not influence RR parameters in patients having rhonchi. Infants and young children with wheeze are more likely to be tachypneic^15^. The WHO suggested that a dose of bronchodilator be administered in a child with tachypnea and wheeze to observe whether the tachypnea improves before consideration of the diagnosis of pneumonia^19^. In addition, we detected no association between RR and body temperature in patients having only cough or cold. Fever of upper respiratory infections might have a limited influence on RR.

In our study, the relationship between body temperature and RR disappeared when chest indrawings were present. The integrated management of childhood illness (IMCI) guidelines define severe pneumonia as chest indrawing with or without fast breathing^4^,^12^.

Oxygen saturation of patients influenced the interaction between body temperature and RR parameters. No relationship was detected in hypoxaemic patients (<95%). WHO guidelines for IMCI also use saturation in the diagnosis and management of childhood disease. In newborns and children, hypoxaemia is associated with increased risk of mortality and is a common complication of bronchiolitis, pneumonia, and asthma^10^. Several studies found that patient's oxygen saturation was associated with a change in clinical management and admission to a hospital in cases^4^,^20^,^21^. Duke et al.^20^ conducted a study that examined the effect of introducing oxygen concentrators on pneumonia-related mortality. It was reported that the detection of hypoxaemia and treating it reduce pneumonia mortality by 35%^20^. Floyd et al. also reported that combining pulse oximetry with the implementation of integrated management of childhood illness treatment guidelines would prevent pneumonia-related deaths annually in the highest-burden countries^21^. Yalcin et al. also evaluated that the agreement between IMCI and final diagnosis in children who presented with a cough at the second and third levels of health institutions^16^. They reported that agreement was found to be high in patients with severe pneumonia and oxygen saturation of <93%.

In this study, we didn't find any relationship between body temperature and RRD in children with oxygen saturation of <95%. There is no gold standard for the definition of pneumonia. Studies have not found a correlation between the height of fever and RRD among children with lower respiratory tract findings^22^, ^23^. In a study, it was found that respiratory rate, adjusted for age, increased by around 2.2 breaths/min per 1°C rise in body temperature without significant contributions from specific age groups^24^. An increase, unadjusted for age, of 2.5 breaths/min per 1°C rise in temperature in two different pediatric populations were also found in studies^25^,^26^. In our study, we found that for every 1°C increase in temperature, the respiratory rate increased by 6.5 minutes in all cases, 8.2/minute in the patients under 12 months of age, 7.1/minute in female, 5.8/minute in male, and 6.6/minute in the patients with prematurity. RRD was 5.7/min with 1°C increase in body temperature.

## Strengths and limitations

This survey enrolled 297 patients and 891 measurements were taken. All cases were followed for a week. Also, for the first time, we calculated individual differences in RR compared to the recovery period. These are the strengths of our work.

As a limitation, our results can not be generalized to other diseases causing fever including urinary tract infections and malignancies. A longitudinal study would be performed toevaluate RRD in other diseases causing fever.

## Conclusion

Respiratory rate should be evaluated by healthcare workers according to the degree of body temperature in children with ARI. However, the interaction between body temperature and respiratory rate could not be observed in cases with rhonchi and low oxygen saturation. The subjective assessment of chest auscultation findings and oxygen saturation may be useful in the evaluation of pneumonia and bronchiolitis risk among children with ARI. Further studies are necessary to detect the interaction between body temperature and RR in other conditions causing fever.
